# Cross-Linked Cellulose Nanocrystal Membranes with Cholesteric Assembly

**DOI:** 10.1021/acs.langmuir.4c01443

**Published:** 2024-06-13

**Authors:** Berk C. İçten, Emre Bukusoglu, P. Zeynep Çulfaz-Emecen

**Affiliations:** Department of Chemical Engineering, Middle East Technical University, Çankaya, Ankara 06800, Turkiye

## Abstract

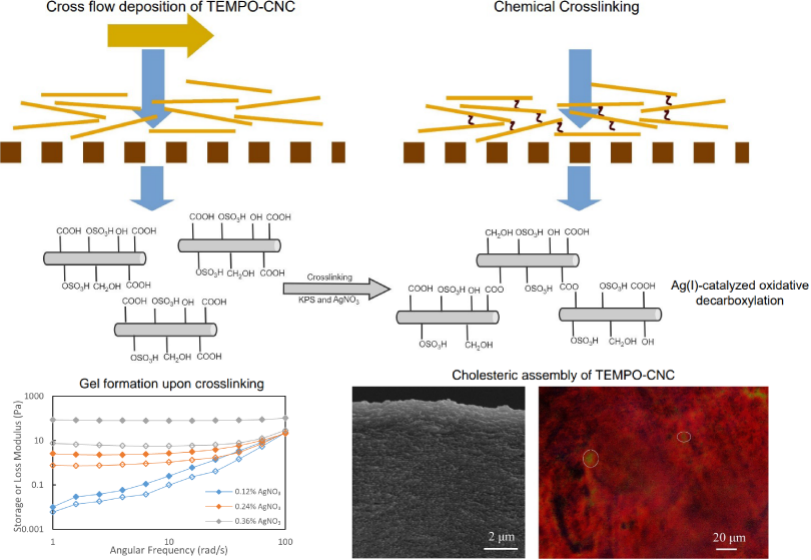

Forming membranes by tangential flow deposition of cellulose nanocrystal (CNC) suspensions is an attractive new approach to bottom-up membrane fabrication, providing control of separation performance using shear rate and ionic strength. Previously, the stabilization of these membranes was achieved by irreversibly coagulating the deposited layer upon the permeation of a high-ionic-strength salt solution. Here, we demonstrate for the first time the chemical cross-linking of carboxyl-containing TEMPO-oxidized CNCs by Ag(I)-catalyzed oxidative decarboxylation and the stabilization of CNC membranes using this post-treatment. Cross-linking of TEMPO–CNCs was first demonstrated in suspension via turbidity, dynamic light scattering, and storage (*G*′) and loss (*G*″) moduli measurements. Membranes were formed by filtering a 0.15 wt % TEMPO–CNC suspension onto a porous support, followed by permeation of the cross-linking solution containing AgNO_3_ and KPS through the deposited layer. Rejection for Blue Dextran with a 5 kDa molecular weight was 95.3 ± 1.9%, 90.6 ± 3.7%, and 95.9 ± 1.0% for membranes made from suspensions of TEMPO–CNC, desulfated TEMPO–CNC. and TEMPO–CNC with 100 mM NaCl, respectively. Suspensions with added NaCl led to membranes with improved stability and cholesteric self-assembly in the membrane layer. Membranes subjected to cross-linking post-treatment remained intact upon drying, while those stabilized physically using 200 mM AlCl_3_ solution were cracked, demonstrating the advantage of the cross-linking approach for scale-up, which requires drying of the membranes for module preparation and storage.

## Introduction

Cellulose nanocrystals (CNC) are nanorods with an aspect ratio of around 10, good mechanical and optical properties, and functionalizable surfaces. They are used for sensor applications,^1^ membrane preparation as filler^2^ or active layer,^3^ photonic optical films,^4^ and gels.^5−7^ Cellulose nanocrystals are conventionally prepared with acid hydrolysis of a cellulose source, such as cotton,^8^ kraft pulp,^9^ or microcrystalline cellulose.^10^ During acid hydrolysis, the cellulose source is added to concentrated hydrochloric acid,^11,12^ sulfuric acid,^13,14^ or phosphoric acid^15^ at a moderate temperature, which leads to the removal of amorphous regions on the cellulose chain while leaving the crystalline regions intact. Furthermore, hydrolysis in sulfuric or phosphoric acid yields colloidally stable suspensions of CNCs due to the charged surface groups grafted onto the crystal surfaces. An attractive feature of CNCs is that their surface can be modified by various functional groups, depending on the desired properties for intended applications. The most common surface modification used is 2,2,6,6-tetramethylpiperidine-1-oxy (TEMPO)-mediated oxidation, which grafts carboxyl groups on the surface of the CNCs.^16^ The addition of carboxyl groups increases the colloidal stability of the CNC suspensions and allows the utilization of further surface modification methods, such as amidation.^17,18^

CNCs show lyotropic liquid crystal properties that can spontaneously self-assemble into a left-handed chiral nematic phase.^19^ Self-assembly of CNCs is a complex process that depends on the size and charge of the particles, additives, suspending medium, and ionic strength. This behavior of CNCs in suspension can also be preserved when dried into a film.^20^ Cholesteric behavior can be characterized in suspension and film form with either fingerprint structures,^21^ Bragg reflections,^22^ or Bouligand arches.^23^ The pitch length, which is defined as the length of one full (2π) twist,^19^ can be modulated with salts,^24^ polymers,^24^ changing nanorod size,^25^ and sonication.^26^ CNCs also show nematic alignment when subjected to shear.

Our group has previously demonstrated the preparation of membranes formed of CNC layers deposited via cross-flow, or tangential flow, filtration onto porous supports.^3,27^ During this process, the concentration of CNCs rejected by the porous support increases due to the permeate drag carrying the CNC suspension toward the wall under the applied transmembrane pressure. Back diffusion from the wall occurs as a result of the concentration difference between the walls and the bulk. When the permeate drag is balanced by back diffusion, no buildup of rejected particles on the wall occurs, a condition described as concentration polarization. With increasing transmembrane pressure at a given cross-flow velocity, the wall concentration increases, and when it reaches the maximum value it can attain, a gel layer starts to form on the surface. Once this happens, further increasing the transmembrane pressure does not increase the permeate flux but increases the thickness of the gel layer that forms, which is called the “limiting flux” phenomenon.^28^ This phenomenon of CNC deposition on a porous support layer forms our approach to CNC membrane fabrication in this study.

In our previous studies, the nematic alignment of CNCs in the flow direction was used to increase the rejection of the membranes.^3^ Furthermore, changing ionic strength was shown to change the membranes’ separation performance, which was attributed to diminished electrostatic repulsion between the nanorods, enabling tighter packing and higher rejection toward the model macromolecules.^27^ In these past studies, the stabilization of the CNC deposit on the membranes was achieved by irreversible coagulation upon increasing the ionic strength using a highly concentrated AlCl_3_ solution after deposition. In view of the potential application of the membranes in a wide variety of conditions, including those of high and variable ionic strength, chemically cross-linking the CNC layer is highly desirable to ensure long-term, stable membrane performance.

In the current study, we apply Ag(I)-catalyzed oxidative decarboxylation as a cross-linking method for TEMPO-oxidized CNCs for the first time in the literature. This reaction has previously been used in cross-linking carboxyl-containing polymers, such as poly(acrylic acid), poly(dimethylacrylamide-*co*-acrylic acid), and poly(*N*-isopropylamide-*co*-acrylic acid) to form hydrogels,^29^ but has never been used to cross-link cellulose nanocrystals or other colloidal materials. Here, we first demonstrate the cross-linking reaction between TEMPO-oxidized CNCs as a function of Ag(I) and persulfate concentration, and then the morphology and separation performance of the membranes thus formed.

## Experimental Methods

### Materials

Dimethyl sulfoxide (DMSO) (99.9% analysis grade) and ethanol (99%) were purchased from Isolab. Sulfuric acid (95–98%), cellulose (cotton linter fibers, medium), cellulose acetate (average molecular weight 50 000), sodium hydroxide (98–100.5%), sodium bromide (99.99% trace metal basis), potassium persulfate (99% ACS grade), 2,2,6,6-tetramethylpiperidine-1-oxy (TEMPO) (99%), and blue dextran (average molecular weight 5 kDa) were purchased from Sigma-Aldrich. Sodium chloride (99%) and hydrochloric acid (fuming 37%) were purchased from Merck. Aluminum chloride hexahydrate (99%) was purchased from Tekkim. Sodium hypochlorite was purchased from Aromel. NanoVan was purchased from Nanoprobes.com.

Reverse osmosis water was used as nonsolvent in nonsolvent-induced phase separation medium and washing. Pure water (18.3 MΩ00b7cm) was used in cross-linking solutions and dialysis.

### Preparation of CNC Suspension

Cellulose was dried at 80 °C for 2 days and stored in a vacuum oven before CNC preparation. Initially, 80 mL of pure water was mixed with 150 mL of sulfuric acid and heated until it reached 45 °C in a water bath. Dried 28.8 g of cellulose was hydrated with 58 mL of pure water in another beaker, which makes the final acid concentration 50% in liquid with the addition of hydrated cellulose. Then, the heated sulfuric acid was mixed with hydrated cellulose. The final solution was left to react in the water bath for 30 min at 45 °C. Finally, the reaction was quenched with cold, pure water by diluting the mixture up to 1 L. CNC suspension was purified from remaining sulfuric acid and formed salts by first decanting and discarding the clear part for 3 days, then dialyzed against pure water until dialysis water conductivity reaches pure water conductivity.

### Desulfation of CNC Suspensions

Desulfation was done by adding concentrated NaOH into CNC suspensions up to a final NaOH concentration of 1 M and left reacting for 180 min at 65 °C. After desulfation, the added NaOH was neutralized first with fuming HCl, and then the pH of the suspension was reduced to 1.5–2 to transform the deprotonated form of CNC to the protonated form for further analysis. Finally, suspensions were cooled and dialyzed against pure water at room temperature until dialysis water conductivity reached pure water conductivity.

### TEMPO Oxidation of CNC Suspensions

Previously prepared CNC solutions were modified to add carboxyl groups to the nanocrystal surfaces via TEMPO oxidation. 0.0295 g TEMPO, 0.324 g NaBr, and 17.6 mL of 4.1% NaClO was added into the suspension per gram of CNC.^17^ The suspension was left to react for 3 h, and pH of the solution was adjusted to 10–10.5 range with the NaOH solution continuously at room temperature. To quench the reaction, 3.7 mL of ethanol was added to convert the unreacted NaClO to chloroform. Then, the pH of the suspension was adjusted to 1.5 to modify deprotonated TEMPO–CNCs to the protonated form for both carboxyl and sulfate half ester groups. Finally, the formed suspension was dialyzed against pure water until dialysis water conductivity reachesd pure water conductivity.

### Membrane Preparation Via Cross-Flow Filtration

TEMPO–CNC membranes were prepared by cross-flow filtration of a TEMPO–CNC suspension onto a support membrane. Support membranes were prepared via phase inversion by casting a solution containing 10 wt % cellulose acetate and 90 wt % DMSO on glass substrate with a casting thickness of 250 μm and immersing into a water bath to complete nonsolvent-induced phase separation. Obtained cellulose acetate membranes were used as the support for the following deposition procedure.

To prepare TEMPO–CNC membranes, 0.15 (w/v) % TEMPO–CNC suspensions with 0 or 100 mM NaCl were used. Before membrane preparation, suspensions were sonicated with a Sonopuls mini20 MS 2.5 probe with 100% amplitude for 20 min. Then membrane preparation was continued by cross-flow deposition of TEMPO–CNC onto the support membranes in a Sterlitech CF042 cross-flow filtration cell. During membrane preparation, time-dependent permeation flux data for the membrane was measured. These measurements were continued until a constant permeate flux was observed at the fixed pressure, and then the filtration pressure was increased. This procedure was performed at two consecutive pressures of 0.8 and 1 bar, where the limiting flux was known to be reached. Then, the membranes were either chemically cross-linked or physically stabilized. Chemical cross-linking was done by permeating a solution of 0.36 wt % AgNO_3_ and 0.08 wt % potassium persulfate (KPS) for 2 h in cross-flow filtration mode at 1 bar trans membrane pressure and room temperature, with the cell covered to eliminate the possibility of silver reduction with light. Physical stabilization was done by permeating 100 mL of a 0.2 M AlCl_3_ solution through the membrane in dead-end mode. Finally, membranes were washed with pure water.

### Characterization Methods

The surface charge characterization of CNC in suspension was done via conductometric titration.^30^ During titration, suspensions with known mass and concentration were added into 120 mL of 1 mM NaCl solution, which is used for obtaining a baseline for conductometry. NaOH with a known concentration (0.75–1.25 mM) was added in 1 mL aliquots, and the conductivity of the sample was measured with each addition. After the addition of a fixed amount of NaOH, recorded conductivity values were corrected for the dilution due to NaOH addition and plotted as NaOH consumption versus corrected conductivity. End points for the titration were found by fitting a line for the decreasing and increasing trends and finding intersection points of those lines (Figure S3). For CNC suspensions, one end point was observed, while TEMPO–CNC suspensions showed two end points. The calculation of the respective charges were calculated with following equations
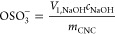
1
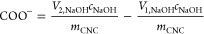
2

In these equations, *m*_CNC_, *V*_*x*,NaOH_, and *c*_NaOH_ are defined as the mass of dried basis CNC or TEMPO–CNC, NaOH volume used to see end points, and concentration of NaOH solution, respectively.

Isoelectric point analyses were performed with the Malvern Zetasizer Ultra in DTS 1070 folded capillary cells. Experiments were done by changing the pH of the suspension with either 0.1 M NaOH or 0.1 M HCl and measuring the zeta potential at pH values between 2 and 12.

The size of cross-linked CNC aggregates in suspension during cross-linking was correlated with turbidity data. Experiments were done by adding KPS and AgNO_3_ to 0.3 (w/v) % TEMPO–CNC suspension and recording turbidity in time using a Hanna Instrument HI88703 turbidimeter. Final hydrodynamic diameter analyses were also done with the Malvern Zetasizer Ultra with Multiple- Angle Dynamic Light Scattering (MADLS) mode with suspensions having a concentration of 0.1 wt %.

Rheological analyses of cross-linked suspensions were done with 2.1% TEMPO–CNC with an Anton Paar MCR 302 rheometer equipped with an Anton Paar CP50–2 cone and plate attachments having a 2.008° cone angle and 0.21 mm minimum spacing at 25 °C in frequency sweep mode for 0.63 to 100 rad/s angular frequencies and 5% constant strain. Gel formation was characterized by measuring storage-to-loss modulus ratio (*G*′/*G*′′).

Pure water permeances of the support and the final membrane were measured in a Sterlitech CF042 cross-flow filtration cell at 1 bar transmembrane pressure. Pure water permeance was calculated with the following equation:
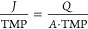
3

In the equation TMP, *Q*, *A*, and *J* were defined as transmembrane pressure (bar), permeate flow rate (L/h), area (m^2^), and flux (L/m^2^h) respectively, while the ratio of *J* and TMP was defined as the permeance.

Rejections were determined by using Blue Dextran (5 kDa, BD5) as a solute with a feed concentration of 0.4 g/L using the same cross-flow filtration cell at 0.4 bar to eliminate concentration polarization with a cross-flow rate of 62 mL/min. Solute concentration was measured using a Shimadzu UV-1601 spectrophotometer at 620 nm. Rejections were calculated with the following equation, where *c*_*p*_ and *c_r_* are permeate and retentate concentrations of BD5, respectively:
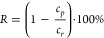
4

Nematic alignment of TEMPO–CNC suspensions was analyzed by flowing the suspensions in a 1.1 mm inner diameter capillary with a New Era NE-300 syringe pump and viewing between crossed polarizers in a Zeiss Axio Scope A1 microscope in transmission mode. Polarized optical images (POM) in reflectance mode were obtained with an Olympus BX53 M microscope to observe Bragg reflections on the final layer.

Scanning electron microscopy (SEM) micrographs were obtained with a TESCAN VEGA 3. Samples were frozen and fractured in liquid nitrogen and sputter-coated with an Au/Pd coating before obtaining micrographs.

Transmission Electron Microcopy (TEM) images were obtained with FEI Tecnai G^2^ Spirit BioTwin CTEM at METU Central Laboratory at 120 kV. Never-dried suspensions with a 0.02 g/L CNC concentration were dropped onto carbon-coated TEM grids, left for 2 min, and wicked with filter paper. After that, NanoVan negative stain was dropped on the grid, left for 30 s, and washed with pure water. All images were analyzed with ImageJ.

The FTIR spectra of films were taken with a Pelkin Elmer Spectrum Two with an UATR attachment. Spectra were reported as an average of 50 scans of wavenumbers in between 600 cm^–1^ to 4000 cm^–1^.

XRD analyses were done with a Rigaku Miniflex in the METU Central laboratory. Samples were supplied as films, and analyses were done with 5°/min scanning rate for angles between 10° and 80°.

## Results and Discussion

### Characterizations of CNC and Tempo–CNC Suspensions

Cellulose nanocrystals isolated from cotton linter using sulfuric acid hydrolysis were analyzed with TEM, which confirmed the rod-like crystals with an average length of 122 ± 38 nm and an average width of 10 ± 2 nm (20 CNCs in 3 images) (Figure S1A). The concentration of sulfate half-esters, which were grafted during hydrolysis, was measured to be 390 ± 30 mmol/kg CNC with conductometric titration (Figure S3A). TEMPO-oxidized CNCs’ dimensions were measured as 113 ± 17 nm in length and 8 ± 2 nm in width (15 CNCs in 3 images) (Figure S1B), close to the unmodified CNCs as expected.^16,31^ The result of TEMPO oxidation was confirmed in the FTIR spectrum, which showed a new peak at 1740 cm^–1^ due to added carboxyl groups on the surface of CNCs (Figure S2). After TEMPO oxidation, the concentration of charged groups was found to be 388 ± 8 mmol/kg CNC of sulfate half esters and 500 ± 40 mmol/kg CNC of carboxyl groups, with two distinct end points observed during conductometric titration^16^ (Figure S3B).

As a third type of CNC containing carboxyl groups with reduced sulfate half-esters, desulfated TEMPO–CNC was prepared. First, desulfation of CNCs was done in alkaline medium, and then the same TEMPO-oxidation procedure was applied to the desulfated CNC suspension. TEM analysis showed an average nanorod length of 148 ± 45 nm and a width of 10 ± 2 nm (21 CNCs in 3 images) (Figure S1C). (. Conductometric analysis showed only a single end point for desulfated TEMPO–CNCs, although the remaining sulfate half-ester content had been measured as 29 ± 8 mmol/kg after desulfation and before TEMPO oxidation (Figure S3C). This is in agreement with previous reports in the literature stating that it is not possible to fully desulfate CNC suspensions in an alkaline medium.^32^ After TEMPO oxidation, carboxyl content was found as 340 ± 40 mmol/kg by assuming sulfate half-ester content to remain constant during TEMPO oxidation (Figure S3D).

**Figure 1:**
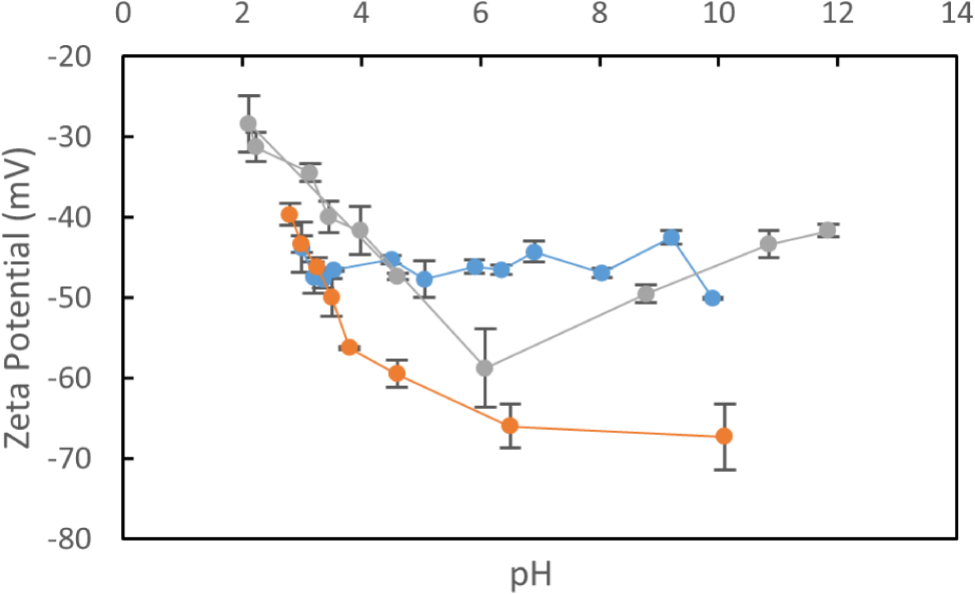
Zeta potential scan results for CNC (blue), TEMPO–CNC (orange), and desulfated TEMPO–CNC (gray) suspensions.

Isoelectric point analyses were performed by measuring the zeta potential of the suspensions as a function of pH. For CNCs, no isoelectric point was observed within the measurable range, as the grafted sulfate half-ester groups are strongly acidic (Figure 1). For TEMPO-oxidized CNCs, zeta potential was lower due to the addition of carboxyl groups at neutral to high pH and decreased with decreasing pH, due to the weak acid properties of the grafted carboxyl groups. The zeta potential of desulfated TEMPO–CNCs was lower than that of TEMPO–CNCs throughout the whole pH range, due to the removal of sulfate groups.

### Cross-Linking of Tempo–CNC in Suspension

TEMPO–CNCs were cross-linked via the Ag(I)-catalyzed oxidative decarboxylation reaction, previously demonstrated in the literature with carboxyl-containing polymers.^29^ In this reaction, Ag(I) and persulfate ions remove grafted carboxyl ions, and the radicals formed via decarboxylation form cross-links. In our study, we first tracked the cross-linking reaction in suspension via turbidity and particle size measurements and later with measurements of storage (*G*′) and loss moduli (*G*″) of concentrated TEMPO–CNC suspensions subjected to different cross-linking conditions.

**Figure 2:**
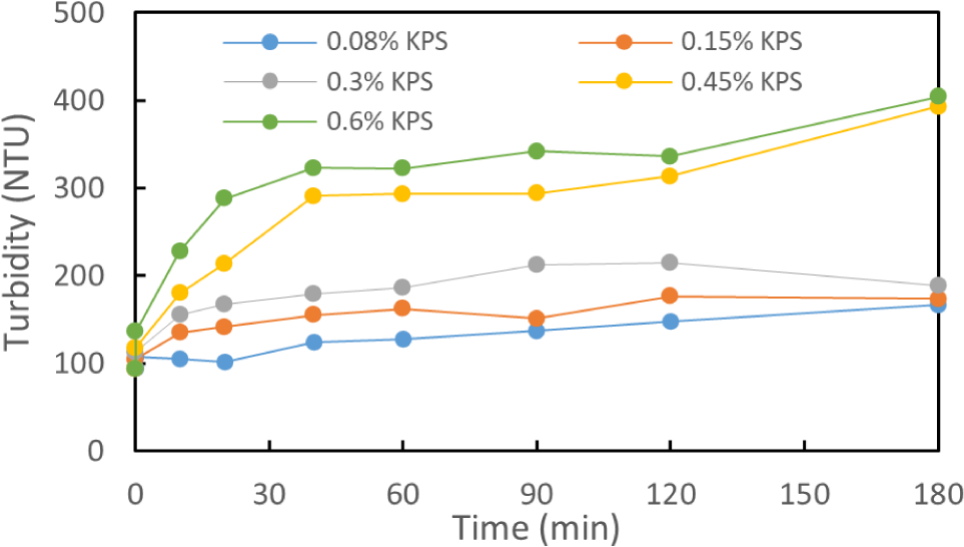
Turbidity changes in 0.3% TEMPO–CNC suspensions during cross-linking in 0.12% AgNO_3_ and 0.08% (blue), 0.15% (orange), 0.3% (gray), 0.45% (yellow), and 0.45% (green) KPS.

Cross-linking of individual nanorods in suspension is expected to result in an increase of average particle size in suspension and a resulting increase in turbidity. Turbidity of 0.3% TEMPO–CNC suspensions during cross-linking was observed in the presence of 0.12% AgNO_3_ and KPS concentrations in the range from 0.08% to 0.60% (Figure 2). Turbidity was observed to increase with increasing reaction time and increasing KPS concentration, indicating an increase in the particle size during the reaction due to cross-linking. The turbidity of the suspension reached a plateau roughly after 2 h, which was selected as the reaction duration in further analyses. Furthermore, when the suspensions were analyzed during the cross-linking reaction, it was observed that the average hydrodynamic radius found from DLS measurements increased from 95 ± 5 nm of the original suspension to 430 ± 3 nm of the cross-linked suspension, indicating the formation of cross-linked networks with size larger than individual nanorods (Figure S4).

During membrane formation, the surface concentration of CNCs is much higher than the bulk concentration as a result of concentration polarization leading to the deposition of the particles.^3^ To gain insight into the structure of the gel layer formed by TEMPO–CNCs on the support layer upon subsequent cross-linking, the rheology of TEMPO–CNC gels was characterized by determining their *G*′ and *G*″ in frequency sweep tests. The tests were done using 2.1% TEMPO–CNC suspensions for a constant strain of 5%. The concentration of AgNO_3_ and KPS used in cross-linking was varied, using a 2-h reaction time in all cases.

**Figure 3:**
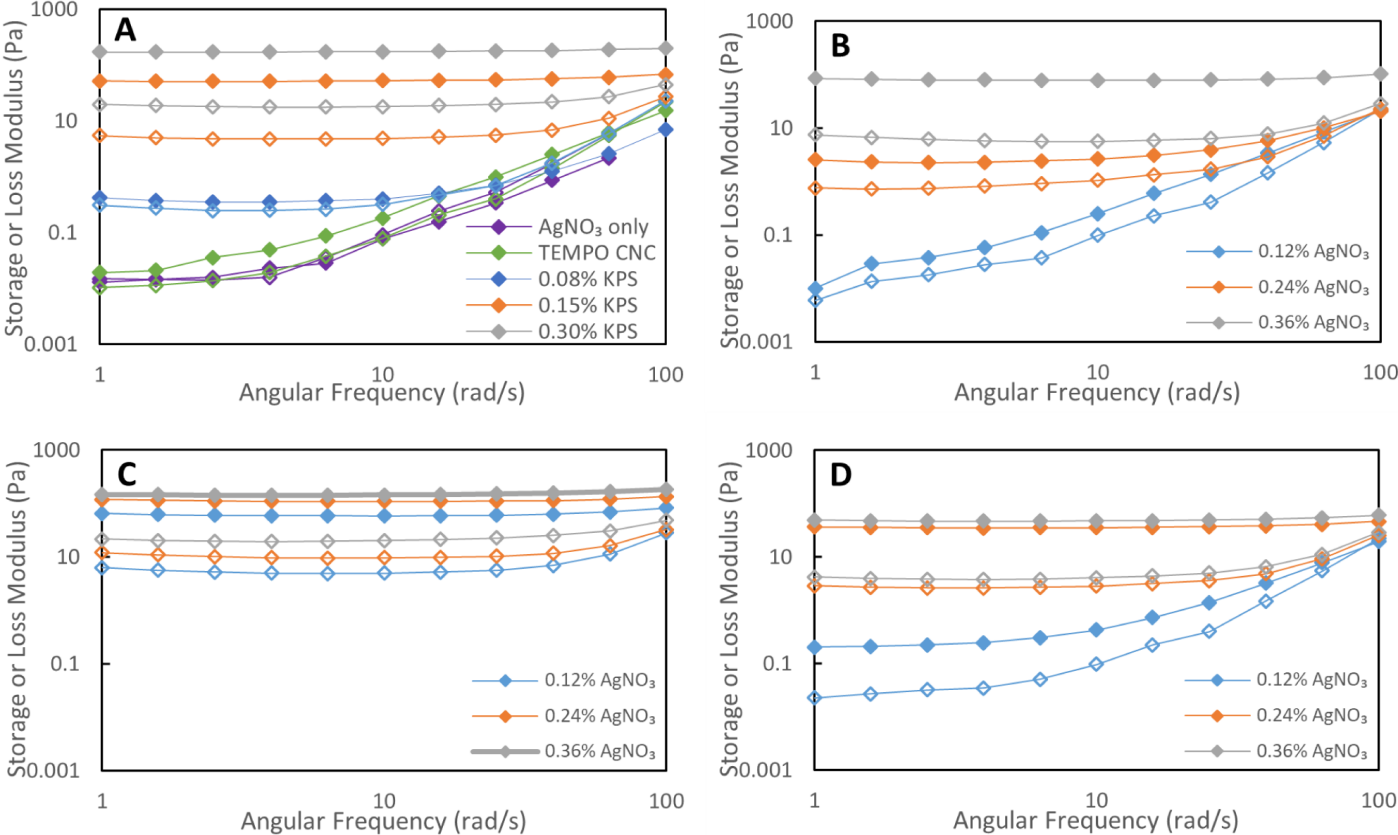
(A) Frequency sweep measurements for 2.1 wt % TEMPO–CNC and 0.12 wt % AgNO_3_ cross-linking after 2 h. The data set labeled TEMPO–CNC shows the suspension without any AgNO_3_ or KPS addition. (B) Frequency sweep results for 2.1 wt % TEMPO–CNC and 0.15 wt % KPS cross-linking with different AgNO_3_ concentrations. (C) Frequency sweep results for 2.1 wt % TEMPO–CNC and 0.08 wt % KPS cross-linking with different AgNO_3_ concentrations. (D) Frequency sweep results for desulfated TEMPO–CNC (2.1 wt %) and 0.08 wt % KPS cross-linking with different AgNO_3_ concentrations. For all figures, filled diamonds show a storage modulus, while empty diamonds show a loss modulus. Measurements were collected at 5% strain amplitude and room temperature.

Frequency sweep measurements on TEMPO–CNC suspension showed that the *G*′ was about twice the *G*″, which suggests the 2.1% TEMPO–CNC suspension is in a weak gel form (Figure 3A). Typically, a material with a *G*′/*G*″ of 10 or higher is generally considered a gel, while a *G*′ being higher than, but of the same order of magnitude as, the *G*′′ indicates a weak gel. Figure 3 shows all the results obtained for frequency sweep tests of cross-linked TEMPO–CNC suspensions. For 0.12 wt % AgNO_3_ concentration, cross-linking experiments were done with no KPS, 0.08 wt %, 0.15 wt %, and 0.30 wt % KPS, which showed increased *G*′ and *G*″ as the concentration of KPS increased. While both moduli increase with increasing KPS, at 0.15 and 0.30 wt % KPS, *G*′/*G*′′ around 10 indicates an increase in elastic behavior and hence gelation. As a result, we can conclude that increasing the KPS concentration leads to increased degree of cross-linking at a constant AgNO_3_ concentration and reaction time. For the application of this cross-linking reaction on the CNC membrane, increasing KPS concentration extensively was not favorable since, as a strong oxidant, it was observed to damage the cellulose acetate support membrane used in this study. Instead, it was decided to keep the KPS concentration constant and increase the AgNO_3_ content. In the previous report of Weng et al., it was mentioned that increasing the AgNO_3_ content also increased the cross-linking density for constant reaction time.^29^ Therefore, further tests were done at constant KPS concentrations of 0.15 and 0.08 wt % and varying AgNO_3_ content as 0.12, 0.24, and 0.36 wt %. For a 0.15 wt % KPS concentration, gel formation was observed at all three AgNO_3_ concentrations (Figure 3B). On the other hand, 0.12 and 0.24 wt % AgNO_3_ concentrations did not lead to gel formation when the KPS concentration was 0.08 wt % (Figure 3C). Therefore, it can be concluded that an increase in both KPS and AgNO_3_ concentrations resulted in better cross-linking for the studied range.

It was hypothesized that the extent of cross-linking would be higher if electrostatic repulsion between particles was diminished, allowing closer packing of particles. To test this, desulfated TEMPO–CNCs were cross-linked with 0.08 wt % KPS at varying AgNO_3_ concentrations (Figure 3D). *G*′/*G*′′ were found as 9.1, 14.3, and 12.5 for 0.12, 0.24, and 0.36 wt % AgNO_3_, while these ratios were 1.7, 3.5, and 11.1 for the nondesulfated counterparts (Figure 3C). Therefore, it was concluded that the desulfation procedure used leads to a higher degree of cross-linking and gel formation. It should be noted that TEMPO-oxidation was done after desulfation; therefore, the carboxyl content was not removed during the addition of alkali and heating. It was previously reported that CNCs can approach closer to each other as the surface charge decreases,^33^ and concentration for gelation decreases with decreased surface charge,^34^ which increases the possibility of cross-linking as the decarboxylation reaction occurs via KPS and AgNO_3_.

In the study of Weng at al., the cross-linking reaction is proposed to proceed via the oxidation of carboxyl groups by Ag(II) species formed from Ag(I) by persulfate and the subsequent formation of acyloxy and alkyl radicals.^35^ After this step of Ag(I)-catalyzed oxidative decarboxylation,^36^ it is hypothesized that the formed radicals cross-link through intermolecular radical combination. This reaction is expected to yield ester linkages between the TEMPO–CNC’s. To demonstrate this, the cross-linked and non-cross-linked TEMPO–CNC membranes were analyzed with FTIR. Because the ester peak overlaps with the carboxyl peak in the spectrum, the carboxyl groups in cross-linked and non-cross-linked TEMPO–CNC layers were converted to carboxylates by placing them in 1 M NaOH. After washing and drying, the FTIR spectra of the cross-linked TEMPO–CNC film were compared with those of the non-cross-linked counterpart. It was observed that, as expected, the carboxyl peaks at 1740 cm^–1^ disappeared and the carboxylate peak at 1620 cm^–1^ became stronger. However, this analysis done on the membranes themselves did not clearly reveal the ester bonds in the cross-linked membrane’s spectrum. Considering that this may be due to the low degree of cross-linking in the membranes, we repeated the analysis on concentrated suspensions of TEMPO–CNC. We carried out the cross-linking reaction for 2 h in 2.25% TEMPO–CNC suspensions with 0.36% AgNO_3_ and 0.30% KPS, after which the suspensions gelated. After the obtained gel was dried into a film and placed in NaOH to convert the remaining carboxyl to carboxylates, the FTIR spectrum showed a clear peak around 1730 cm^–1^, suggesting the formation of ester linkages (Figure S5).

### Membrane Preparation Via Cross-Flow Deposition of Tempo–CNC Suspensions and Chemical Cross-Linking

TEMPO–CNC suspensions were used in the formation of membranes via cross-flow deposition on a porous support. Prepared supports showed pure water permeance (PWP) of 871 ± 480 L/m^2^hbar on average and Blue Dextran (5 kDa) rejection below 52%. As it was observed that KPS damages the support, evidenced by an order-of-magnitude increase in PWP of support, it was decided to use a low KPS and high AgNO_3_ concentration of 0.08 and 0.36 wt %, respectively, for cross-linking TEMPO–CNC membranes.

0.15% TEMPO–CNC suspension was used as the feed in cross-flow deposition. Gel formation caused by concentration polarization was used as the membrane preparation method, similar to our previous studies^3,27^ (Figure S6). For a 62 mL/min cross-flow rate, corresponding to a wall shear rate of 33 s^–1^, the limiting flux was found to be 15.0 ± 1.4 L/m^2^ h. After CNC deposition, membranes were cross-linked by permeating a 0.36% AgNO_3_–0.08% KPS solution for 2 h through the TEMPO–CNC deposited support at a transmembrane pressure of 1 bar. The BD5 rejection of the membranes formed under these conditions was 95.3 ± 1.9%.

The limiting flux during membrane formation with desulfated TEMPO–CNC under identical conditions was found to be 13.8 ± 1.1 L/m^2^ h, and the BD5 rejection was 90.6 ± 3.7%. The resistances of the CNC layers on top of the porous supports were calculated using Darcy’s law and the resistances-in-series model, with the PWP of the support layer giving the support resistance and the PWP of the CNC membrane giving the sum of the support resistance and the CNC-deposit layer. The values are 2.9 ± 0.4 × 10^–13^ m^–1^ and 3.2 ± 0.4 × 10^–13^ m^–1^, for TEMPO–CNC and desulfated TEMPO–CNC membranes, respectively. The limiting flux, CNC layer resistance, and BD5 rejections are close within the error margins of at least three membranes fabricated. However, on average, the desulfated TEMPO–CNC membrane shows a lower limiting flux, which implies a thicker deposit layer as the membrane formation was carried out at the same pressures for both membranes. The higher average resistance can be attributed to the increased thickness as well as the decreased porosity of the deposit layer, which can be expected due to reduced electrostatic repulsion between the nanorods bearing a lower charge density. The lower average rejection under these conditions may then be related to the lower negative charge on the membranes caused by desulfation. The dye loading of BD5 is reported as 0.01–0.08 mmol/g by the manufacturer, which may result in electrostatic repulsion between the membranes and the dextran chains.

The membranes formed were observed to crack easily after fabrication, which was attributed to stress formation within the relatively thick CNC layer (measured to be on the order of 10 μm). To reduce the stress, 100 mM NaCl was added to the feed TEMPO–CNC suspension, and membranes were formed by deposition on the supports using the same cross-flow rate. The limiting flux was found to be 7.15 ± 2.36 L/m^2^h, distinctly lower than when no salt was used, and the CNC layer resistance was 3.5 ± 1.0 × 10^–13^ m^–1^. The BD5 rejection of this membrane was found to be 95.9 ± 1.0%. The membranes deposited in 100 mM NaCl show Bragg reflections while wet, with the color remaining unchanged when the membranes angle with respect to the polarizers were changed, indicating the formation of cholesteric self-assembly within the TEMPO–CNC layer, with pitch sizes in visible wavelength range during deposition even though the estimated wall shear rate of 33 s^–1^ is expected to cause nematic alignment of the nanocrystals.^3^

**Figure 4:**
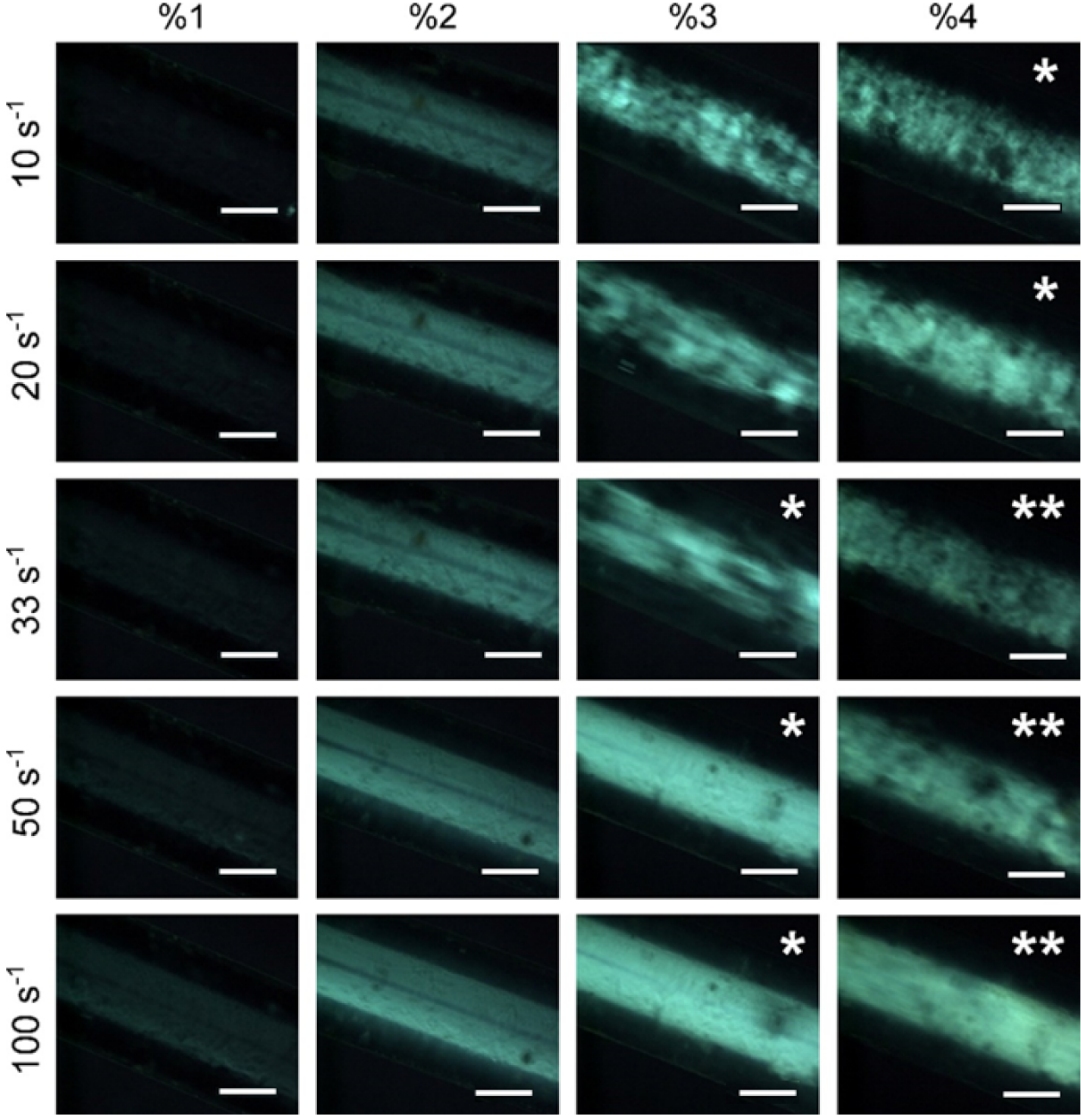
Polarized optical microscopy images of TEMPO–CNC suspensions flowing in the capillary. Columns show 1–4 wt % TEMPO–CNC in suspension, and the rows show 10–100 s^–1^ wall shear rate. Images are taken between crossed polarizers in transmission mode. The scale bar shows 1 mm. Images containing a single asterisk describe that the image were obtained with half exposure, while the double asterisks describe that the images were obtained with quarter exposure. Brightness shows light transmission through crossed polarizers, indicating the alignment of CNCs.

Figure 4 shows transmission-mode polarized optical microscopy images for TEMPO–CNC suspensions at different concentrations flowing in a capillary at different flow rates corresponding to wall shear rates between 10 and 100 s^–1^. All concentrations showed birefringence caused by nematic alignment at shear rates starting from 10 s^–1^. Images of suspensions with increased TEMPO–CNC concentrations show the formation of aggregates at low shear rates. These aggregates appeared to be mostly broken when the shear rate increased, and highly birefringent and clear suspensions were observed in the POM.

The presence of aggregates within the nematic shear-aligned suspensions is in line with the observation of Bragg reflections, indicating cholesteric self-assembly. In our previous study on forming membranes using CNC suspensions at varying salt concentrations, we concluded that salt-induced aggregation and tactoid formation occurred within the mobile yet concentrated mass transfer boundary layer next to the deposited membrane on the wall. The occurrence of tactoids at low shear rates and TEMPO–CNC concentrations, as demonstrated in Figure 4, and the Bragg reflections of the membranes indicate that these tactoids are dominant in the resulting membrane structure forming on the porous support.

**Figure 5:**
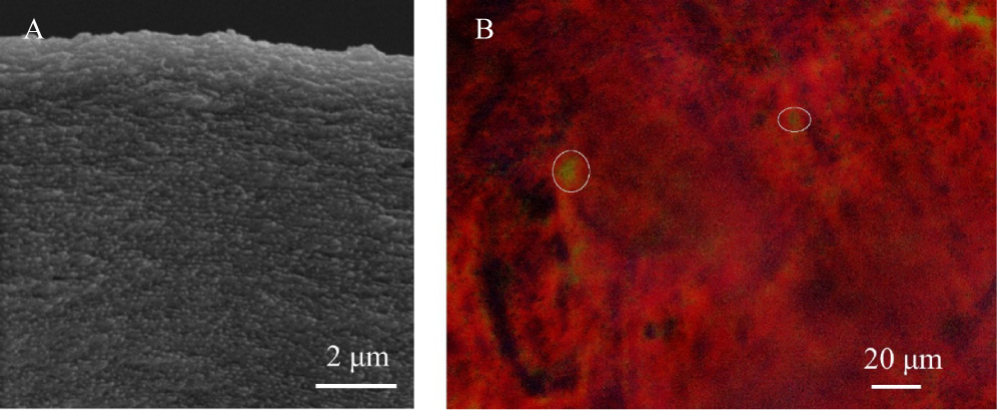
Cross-section SEM image of the dried membrane (A) and the polarized optical microscope image of the wet membrane between crossed polarizers (B). Representative tactoid regions were showed in the figure. Full membrane cross-section showing the support layer is given in Figure S8.

The membranes were also analyzed with a polarized optical microscope while wet to observe reflected light, which showed tactoids as different colored domains, such as green colored regions in Figure 5B. After analysis, membranes were dried, and SEM images were collected from membrane cross sections. Figure 5A shows SEM and reflection mode polarized optical micrographs of cross sections of a TEMPO–CNC membrane fabricated with a 0.15 wt % TEMPO–CNC suspension in 100 mM NaCl, using a 62 mL/min cross-flow rate corresponding to 33 s^–1^ wall shear rate. The SEM image shows Bouligand structures which are characteristic for cholesteric CNC deposit layers. The pitch length in this image is 257 ± 60 nm. When the membrane is allowed to dry under the microscope, the change in color, indicating a decrease in pitch size, was observed (Figure S7).

**Figure 6:**
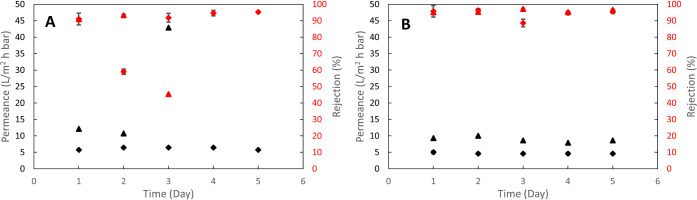
Long-term pure water permeance and blue dextran (5 kDa) rejection for the TEMPO–CNC membrane without salt addition during TEMPO–CNC depositions (A) and the TEMPO–CNC membrane with 100 mM NaCl addition during deposition (B). Diamond data points represent physically stabilized membranes, and triangles represent chemically cross-linked membranes. Error bars indicate the standard deviation of measurements taken at steady-state on each day.

To observe the long-term stability of the membranes, filtration tests were carried out by continuous pure water permeation for 5 days and daily blue dextran rejection measurements. Cross-linked membranes prepared with TEMPO–CNC suspensions containing no salt were found to be stable for 2 days, while the TEMPO–CNC layer delaminated at the third day. While the TEMPO–CNC layer itself was intact, the adhesion to the substrate was poor, which can be improved by the surface modification of the support. For comparison, AlCl_3_-stabilized membranes were also prepared with the method previously mentioned in the literature using the same cross-flow velocity of 62 mL/min.^3^ AlCl_3_-stabilized membranes showed 5 days of stable pure water flux and BD5 rejection (Figure 6A).

Membranes made using TEMPO–CNC suspensions containing 100 mM NaCl and either chemically cross-linked or physically stabilized using AlCl_3_ were stable during 5 days of continuous operation with constant pure water permeance and BD5 rejection. During the stability tests, pure water permeance and BD5 rejection were 4.7 ± 0.2 L/m^2^hbar and 94.2 ± 2.8%, respectively, for the physically stabilized membrane, while these were 8.9 ± 0.7 L/m^2^hbar and 95.9 ± 1%, respectively, for the chemically cross-linked membranes during a period of 5 days (Figure 6B).

**Figure 7:**
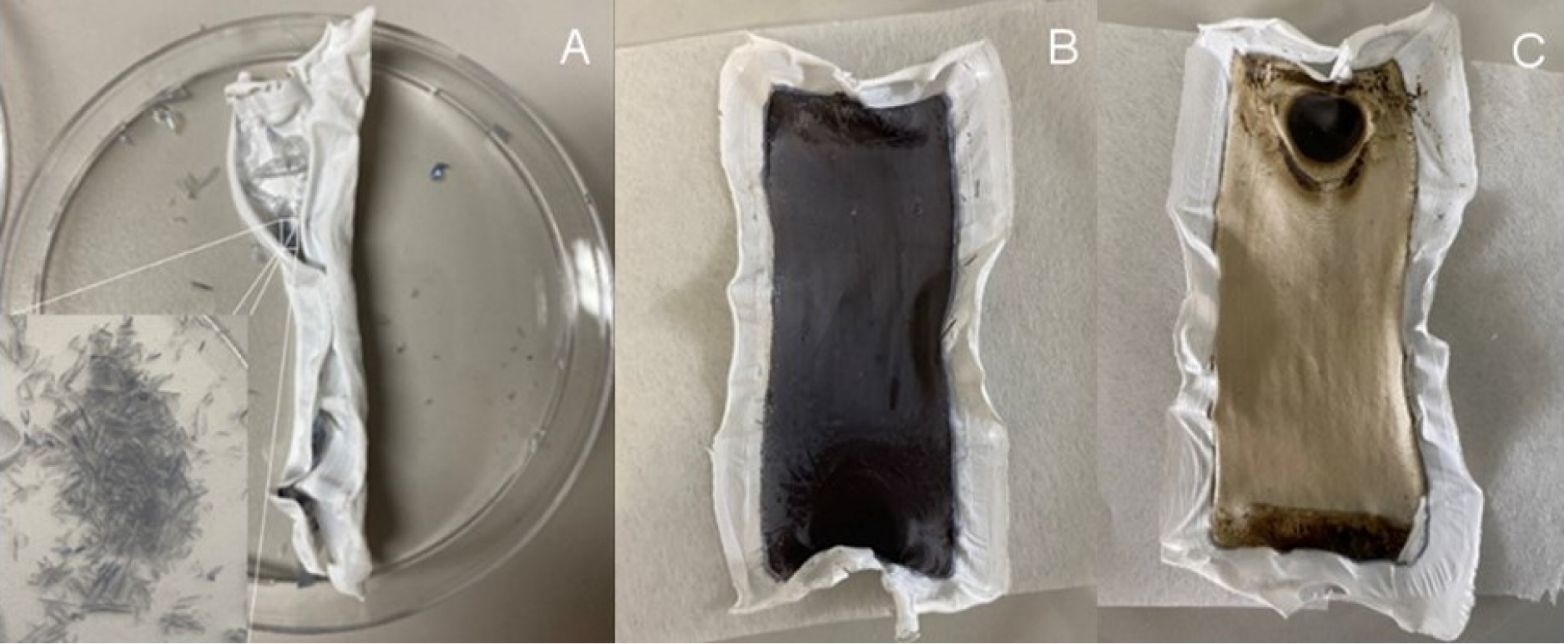
Photographs of dried membranes: (A) AlCl_3_-stabilized membrane and delaminated layer (inset), (B) cross-linked membrane, and (C) cross-linked membrane after Na_2_S_2_O_3_ treatment.

Figure 6 shows photographs of the membranes after drying. It was observed that the membranes stabilized using AlCl_3_ cracked and delaminated upon drying, while the chemically cross-linked membranes were intact and attached to the support layer (Figure 7A). This is important for the final use of the membranes, which will most likely need to be dried for potting inside the modules and storage. For the cross-linked membranes, black colored precipitate formation was observed after removal of the membrane from the filtration cell. These black colored precipitates are thought to be caused by silver impurities formed during the cross-linking reaction and adsorbed on the membrane surface (Figure 7B). XRD analysis of the membrane surface showed silver (face-centered cubic) and silver chloride as the major crystalline phases other than cellulose, which is in the cellulose-I structure as expected (Figure S9). By filtering Na_2_S_2_O_3_ solution through these membranes, it was observed that these impurities could be partly removed, which suggests that silver oxide is also present, as this is known to react with sodium thiosulfate to form soluble silver salts^37^ (Figure 7C). This also demonstrates that the precipitated silver phases can be solubilized and washed off the membrane.

## Conclusion

Membranes were fabricated by depositing TEMPO-oxidized CNCs onto porous support layers via cross-flow filtration and subsequent cross-linking using Ag(I)-catalyzed oxidative decarboxylation of TEMPO–CNCs. This is the first demonstration of chemical stabilization of membranes formed using CNCs as examples of colloidal nanorods, which were previously shown to form ultrafiltration membranes whose rejection properties could be tuned by varying shear rates, ionic strengths, and pH.^3,27^ Evidence of the cross-linking reaction was obtained from increasing turbidity and hydrodynamic radius of suspensions as a function of increasing reaction time and *G*′/*G*′′ of more concentrated suspensions subjected to the cross-linking reaction at different concentrations of AgNO_3_ and KPS. It was observed that increasing concentrations of both AgNO_3_ and KPS increased the extent of cross-linking, and desulfated TEMPO–CNC suspensions showed gel properties at lower AgNO_3_ concentrations, possibly due to the reduced electrostatic interactions between the nanorods allowing closer contact and a higher possibility for the cross-linking reaction.

The membranes formed by depositing TEMPO–CNC suspensions followed by cross-linking were found to crack easily, which was attributed to internal stresses forming in the CNC film upon cross-linking. These could be diminished by adding NaCl to the suspensions, which also resulted in cholesteric self-assembly in the membranes. The formation of cholesteric aggregates in the flowing suspension was demonstrated by viewing the flow of suspensions of varying concentrations under crossed polarizers, and the presence of a cholesteric assembly was shown by SEM images and polarized optical microscope images showing visible color, which changed upon drying.

All membranes demonstrated higher than 90% rejection toward Blue Dextran of 5 kDa molecular weight (BD5). The long-term stability was investigated in continuing water permeation tests with daily BD5 rejection measurements. It was demonstrated that membranes coated with CNC suspensions containing 100 mM NaCl were stable for 5 days when stabilized by chemically cross-linking or by permeating 200 mM AlCl_3_ as a physical stabilization approach we previously used. Upon drying, the physically stabilized membranes cracked and delaminated from the support, while chemically cross-linked membranes remained intact. This is a promising result for the application of CNC membranes in conditions of varying ionic strength, as the chemically cross-linked layer is expected to remain stable in the presence of salts.

## Supporting Information Available

TEM micrographs of CNCs (Figure S1); FTIR spectra of CNCs (Figure S2); representative conductometry measurements of CNCs (Figure S3); DLS analyses of cross-linked CNC suspensions (Figure S4); FTIR spectra of TEMPO–CNC and cross-linked TEMPO–CNC layers (Figure S5); representative TEMPO–CNC membrane preparation procedure showing permeate flux as a function of time (Figure S6); reflection mode polarized optical microscope images of CNC layers during drying (Figure S7); full membrane cross-section showing support membrane and CNC layer (Figure S8); XRD patterns of TEMPO–CNC and cross-linked TEMPO–CNC membranes (Figure S9) (PDF)
