# Supplementary material for: Cross-Linked Cellulose Nanocrystal Membranes with Cholesteric Assembly
Source: Langmuir. 2024 Jun 13;40(25):13247–55. doi: 10.1021/acs.langmuir.4c01443 (PMC11210283; doi:10.1021/acs.langmuir.4c01443)
Supplement: Supplementary file 1 — la4c01443_si_001.pdf [file la4c01443_si_001.pdf]

Supplementary Information

Crosslinked cellulose nanocrystal membranes with cholesteric assembly

B. C. İçten, E. Büküşoğlu, P. Z. Çulfaz-Emecen

Department of Chemical Engineering, Middle East Technical University, Dumlupınar Bulvarı No. 1, Çankaya, Ankara, 06800 Turkiye

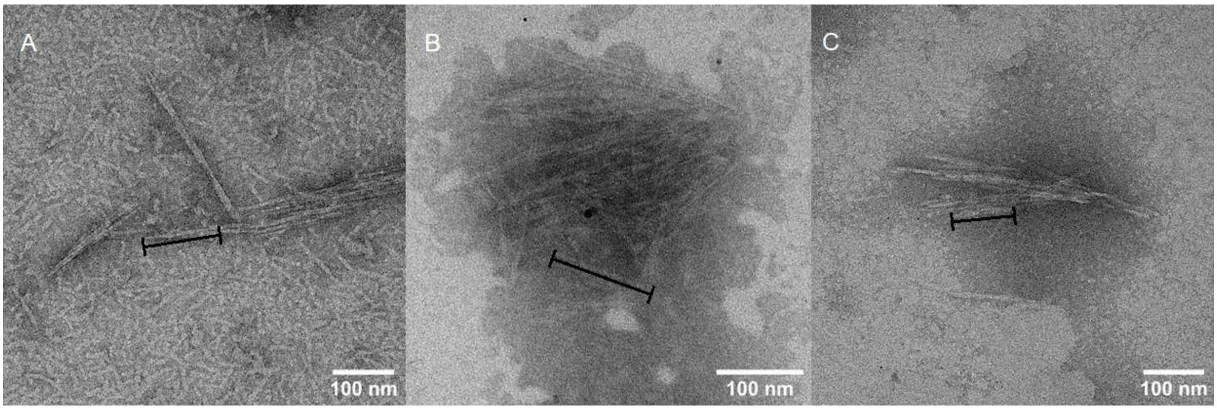

Figure S1. TEM micrographs of (A) CNC, (B) TEMPO-CNC, and (C) desulfated TEMPO-CNC.

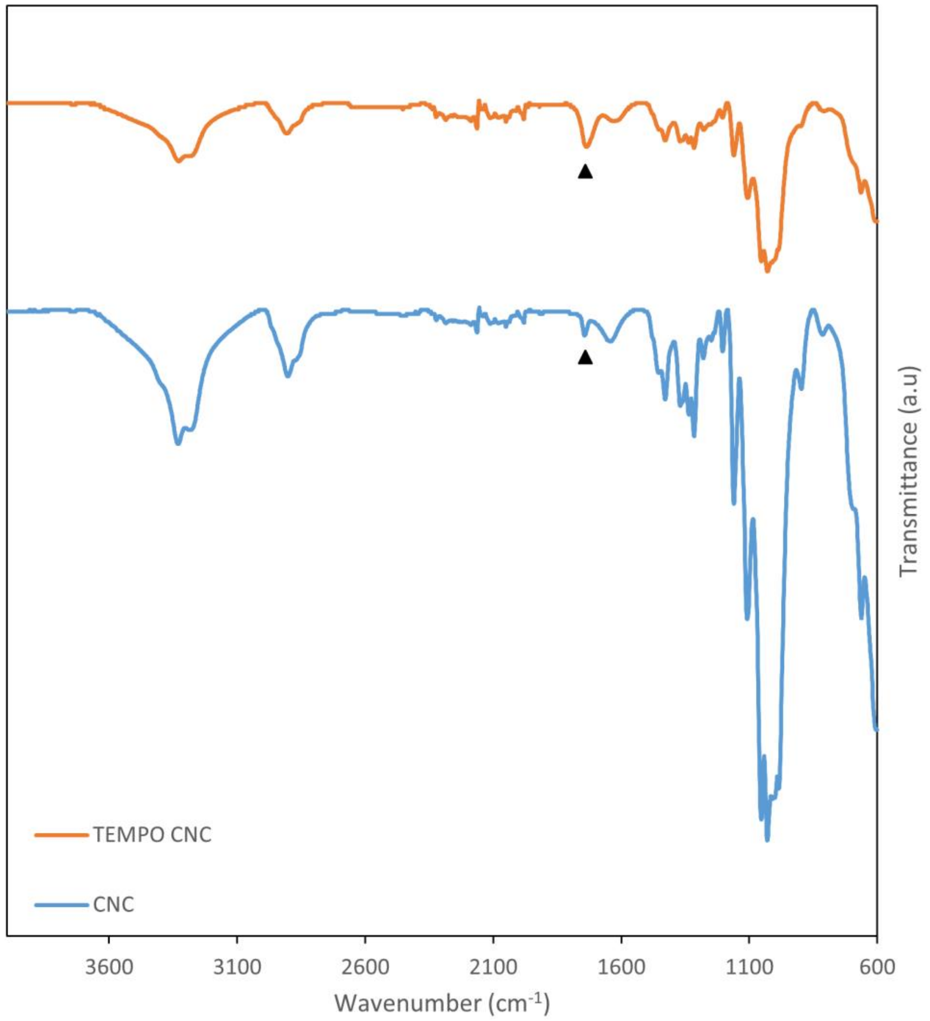

Figure S2: FTIR spectra for CNC and TEMPO CNC.

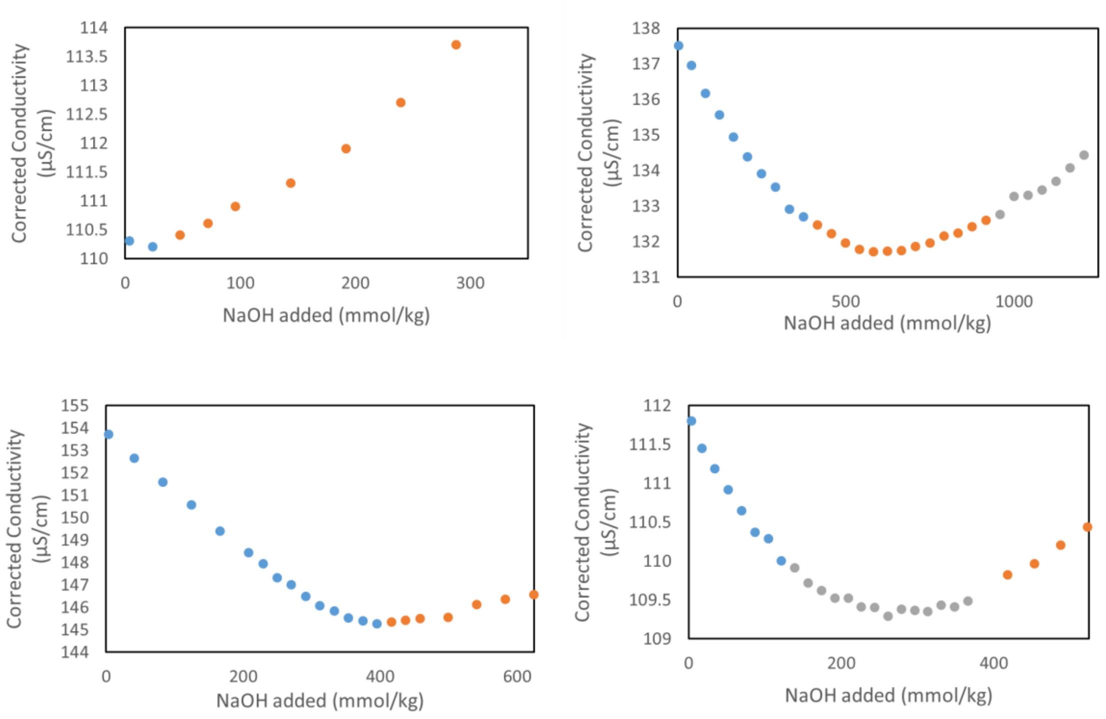

Figure S3. Representative conductometry results of CNC (A), TEMPO CNC (B), desulfated CNC (C) desulfated TEMPO CNC (D).

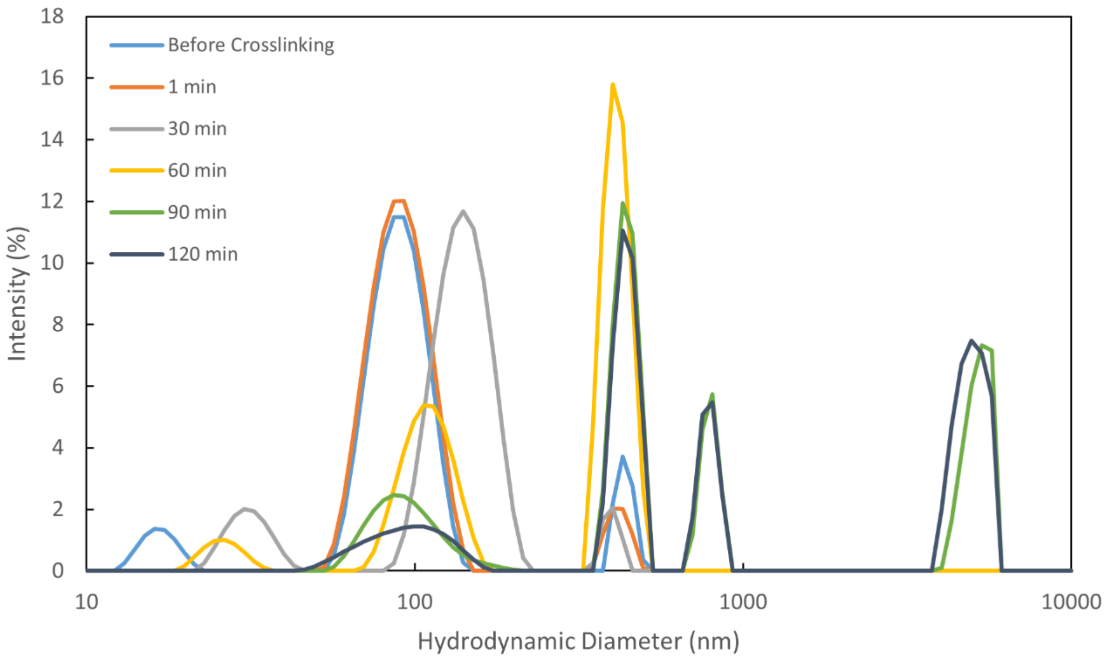

Figure S4: Intensity averaged hydrodynamic diameter distribution at different times of crosslinking of 0.5 wt.% TEMPO CNC suspensions.

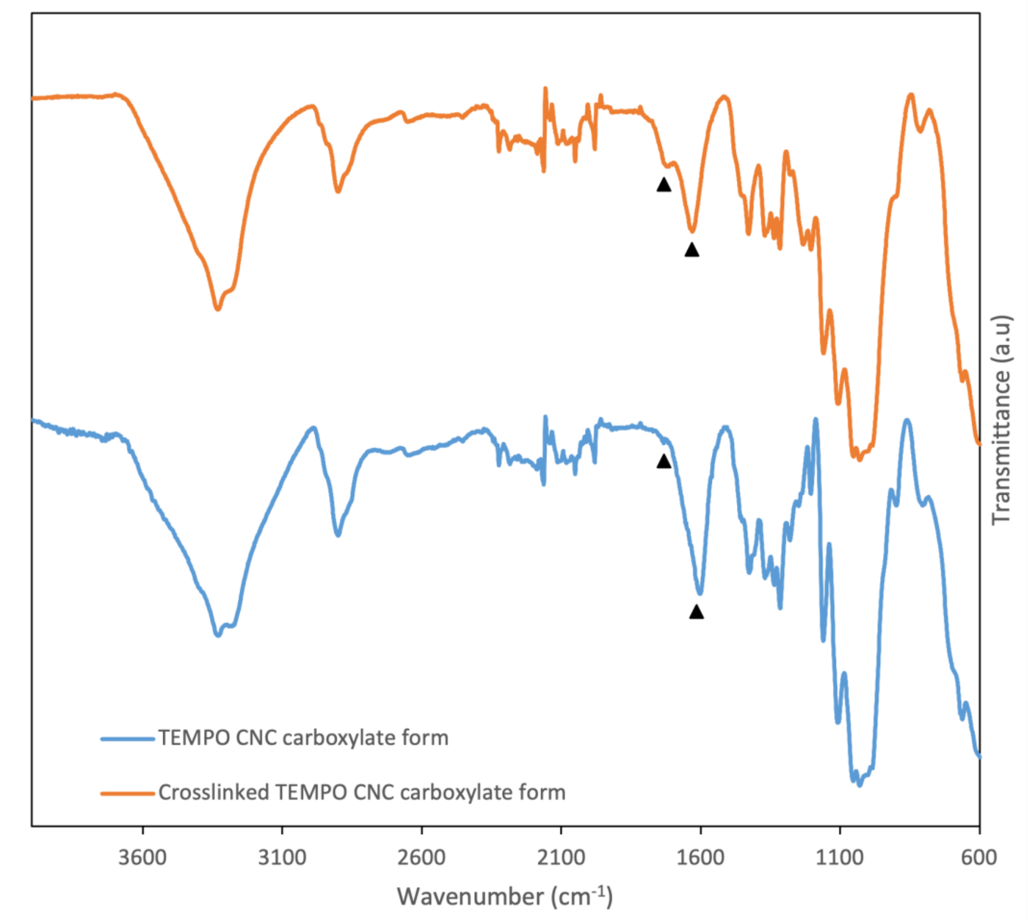

Figure S5. FTIR spectra of TEMPO-CNC and crosslinked TEMPO-CNC in carboxylate form.

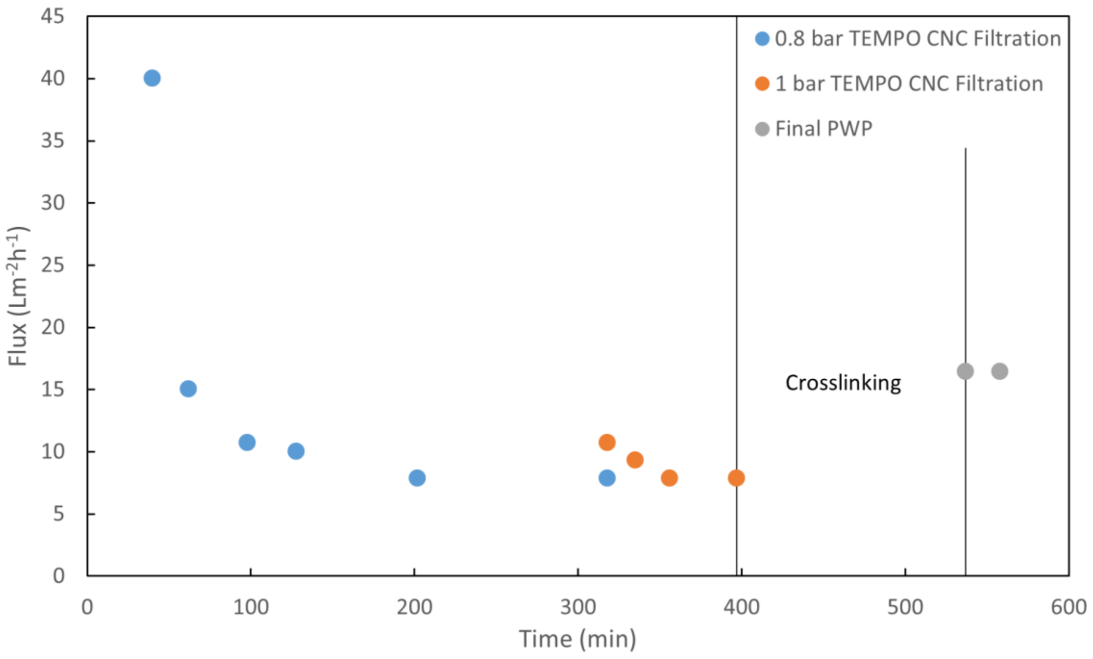

Figure S6. Representative TEMPO CNC membrane preparation procedure. After TEMPO CNC deposition at 0.8 bar and 1 bar, crosslinking solution was permeated through the membrane, followed by pure water permeance measurement.

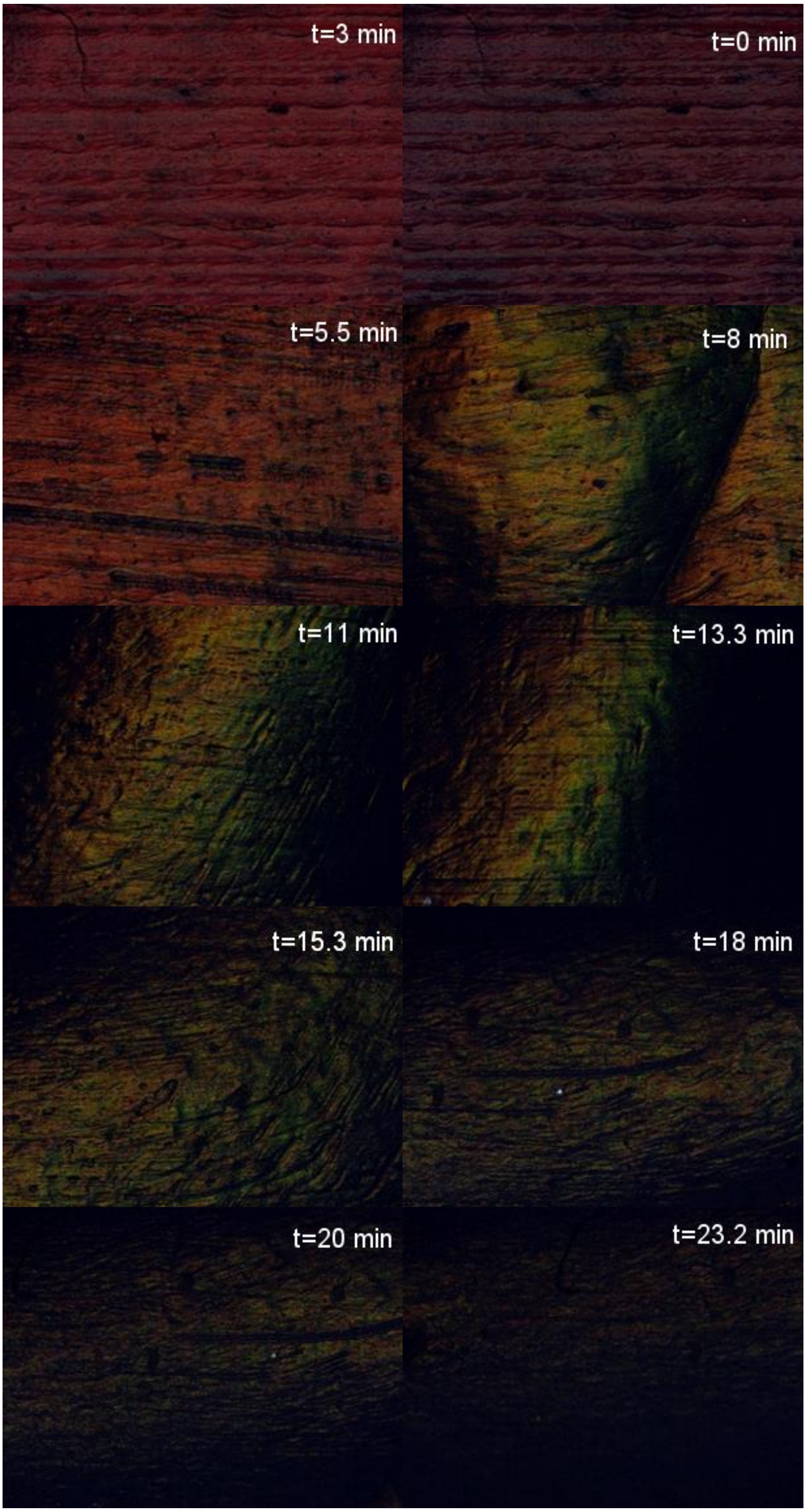

Figure S7. Reflection mode polarized optical microscope image of wet TEMPO-CNC layer during drying.

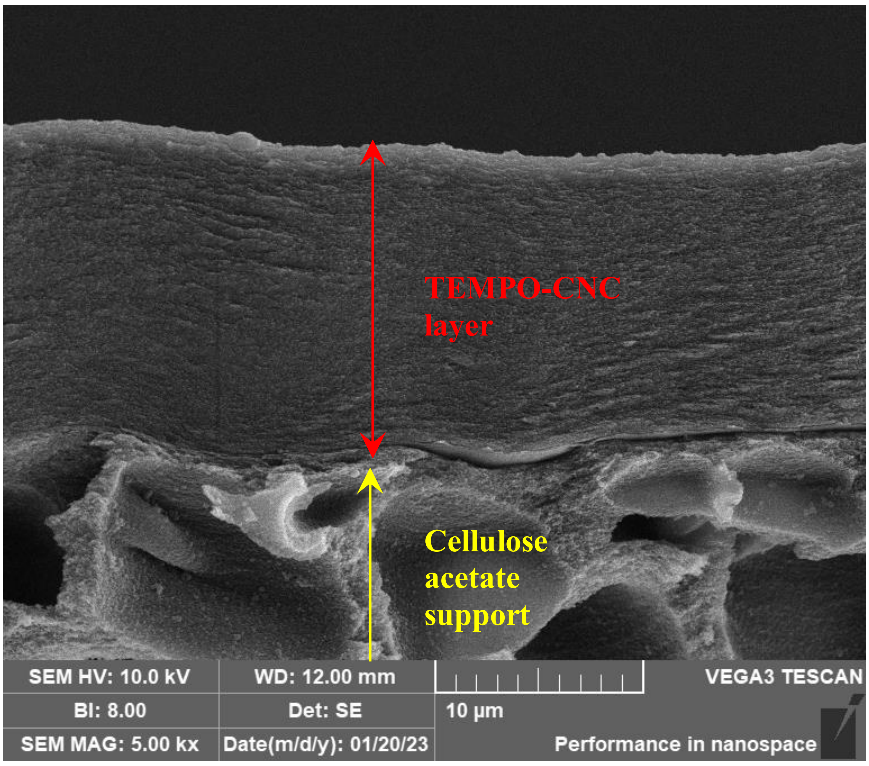

Figure S8. Cross section SEM image of composite membrane showing the cellulose acetate support and the TEMPO-CNC layer

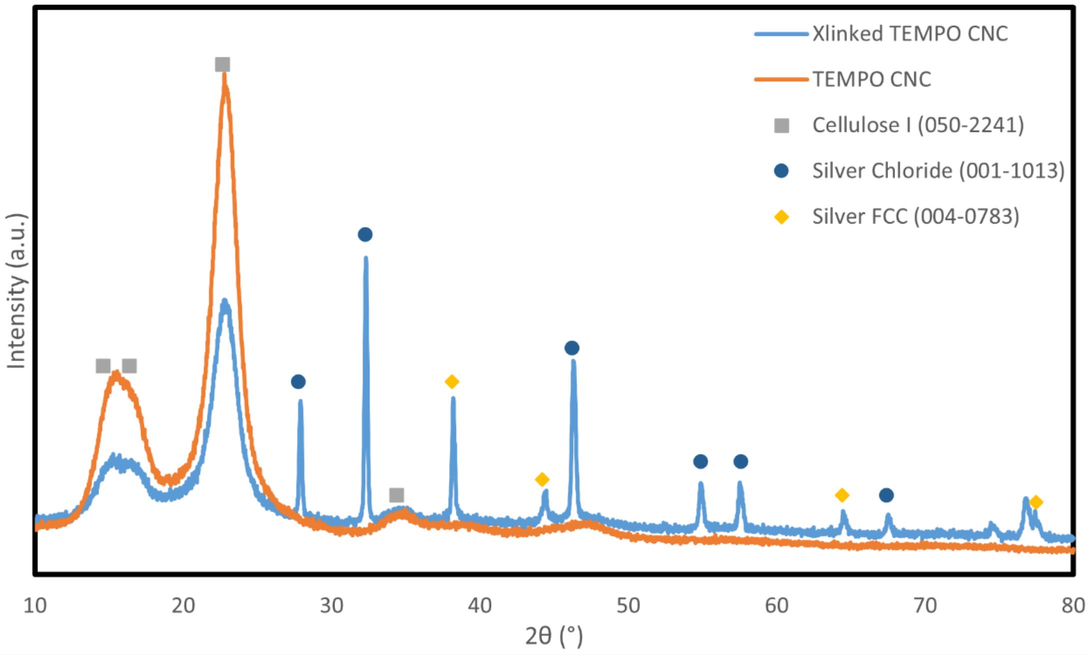

Figure S9: XRD patterns of the TEMPO-CNC and crosslinked TEMPO-CNC membranes. Standard XRD patterns are obtained from ICDD cards, whose numbers are given in parentheses.

References

Boruah, P., Gupta, R., & Katiyar, V. (2023). Fabrication of cellulose nanocrystal (CNC) from waste paper for developing antifouling and high-performance polyvinylidene fluoride (PVDF) membrane for water purification. *Carbohydrate Polymer Technologies and Applications*, *5*. https://doi.org/10.1016/j.carpta.2023.100309

Dhoondia, Z. H., & Chakraborty, H. (2012). *Lactobacillus Mediated Synthesis of Silver Oxide Nanoparticles Regular Paper*. www.nbi.nlm.nih.gov/BLAST

Janardhanan, R., Karuppaiah, M., Hebalkar, N., & Rao, T. N. (2009). Synthesis and surface chemistry of nano silver particles. *Polyhedron*, *28*(12), 2522–2530. https://doi.org/10.1016/j.poly.2009.05.038

Siddiqui, M. R. H., Adil, S. F., Nour, K., Assal, M. E., & Al-Warthan, A. (2013). Ionic liquid behavior and high thermal stability of silver chloride nanoparticles: Synthesis and characterization. *Arabian Journal of Chemistry*, *6*(4), 435–438. https://doi.org/10.1016/j.arabjc.2013.05.005
